# Highly Sensitive β-Lactoglobulin Fluorescent Aptamer Biosensors Based on Tungsten Disulfide Nanosheets and DNase I-Assisted Signal Amplification

**DOI:** 10.3390/molecules28083502

**Published:** 2023-04-16

**Authors:** Yuying Wang, Sisi Chen, Wanmei Chen, Jingjing Wang, Kun Li, Chengyi Hong, Kailong Zhang, Quansheng Chen

**Affiliations:** 1 College of Ocean Food and Biological Engineering, Jimei University, Xiamen 361021, China; 2 School of Life Sciences, Longyan University, Longyan 364012, China; 3 Fujian Provincial Key Laboratory of Food Microbiology and Enzyme Engineering, Xiamen 361021, China; 4 Fujian Provincial Key Laboratory for the Prevention and Control of Animal Infectious Diseases and Biotechnology, Longyan 364012, China; 5 Fujian Province Universities Key Laboratory of Preventive Veterinary Medicine and Biotechnology, Longyan University, Longyan 364012, China

**Keywords:** β-lactoglobulin, aptamer, WS_2_ nanosheets, DNase I

## Abstract

β-lactoglobulin (β-Lg) is a protein found in milk that can cause severe allergic reactions, including rash, vomiting, and diarrhea. Thus, it is crucial to develop a sensitive β-Lg detection method to protect people who are susceptible to allergies. Here, we introduce a novel and highly sensitive fluorescent aptamer biosensor for detecting β-Lg. First, a fluorescein-based dye (FAM)-labeled β-lactoglobulin aptamer (β-Lg aptamer) is adsorbed on the surface of tungsten disulfide (WS_2_) nanosheets via van der Waals forces, resulting in fluorescence quenching. When β-Lg is present, the β-Lg aptamer selectively binds to β-Lg, causing a conformational change in the β-Lg aptamer and releasing it from the surface of WS_2_ nanosheets, which restores the fluorescence signal. Simultaneously, DNase I in the system cleaves the aptamer bound to the target, producing a short oligonucleotide fragment and releasing β-Lg. The released β-Lg then binds to another β-Lg aptamer adsorbed on WS_2_, initiating the next round of cleavage, resulting in significant amplification of the fluorescence signal. This method has a linear detection range of 1–100 ng mL^−1^, and the limit of detection is 0.344 ng mL^−1^. Furthermore, this approach has been successfully used for detecting β-Lg in milk samples with satisfactory results, providing new opportunities for food analysis and quality control.

## 1. Introduction

Food allergy is an adverse reaction that occurs when the immune system responds to a specific food after repeated exposure, which can result in various skin and gastro-intestinal problems and, in severe cases, can be life-threatening. Thus, food allergy has become a significant global food safety issue [1,2]. The Food and Agriculture Organization of the United Nations has identified cows’ milk as one of the eight food allergens [3]. Cows’ milk protein allergy (CMPA) is a common food allergy among infants and children, which can cause allergic rhinitis, asthma, eczema, diarrhea, gastrointestinal bleeding, and other symptoms [4]. CMPA is mainly caused by αs1-casein, α-lactalbumin (α-La), and β-lactoglobulin (β-Lg) as common allergens [5]. β-Lg, which exists mainly as a noncovalently linked dimer and contains 162 amino acid residues with a molecular weight of 18 kDa, is particularly significant. It accounts for 50% of whey protein and 12% of total protein in cows’ milk, with an average concentration of 2–4 g/L [6]. Epidemiological studies suggest that 2–3% of infants and children experience allergic reactions to proteins in cows’ milk, with around 82% of cow milk allergy patients being allergic to cow’s milk β-Lg [7]. Furthermore, β-lactoglobulin is widely employed in food processing due to its high nutritional value, hypotensive, antioxidant, anti-microbial activity, and immunomodulatory effects, increasing the risk of exposure for patients with cow’s milk allergy [8].

Numerous analytical methods have been developed to detect β-Lg, which can be classified into three main categories based on the following detection principles: polymerase chain reaction assays based on allergenic DNA, chromatographic separation method based on allergenic proteins, and immunoassay based on allergenic proteins. However, bovine milk typically lacks allergen-related DNA, so chromatographic and immunological methods are primarily used [9,10,11]. Due to the accuracy requirements of the instrument, these approaches often require rigorous pretreatment procedures, long analysis times, and special laboratory skills [12]. In contrast, immunoassays based on antibody-antigen recognition have significantly enhanced the analysis of trace substances, with traditional methods mainly using enzyme-linked immunosorbent assays [13]. As technology advances, nanomaterials such as magnetic nanobeads, colloidal gold, quantum dots, graphene, and carbon nanotubes can be used to increase detection rates and sensitivity [14]. Although this approach has high specificity and sensitivity, it is expensive, and maintaining antibody stability can be challenging. Furthermore, the quality differences in antibody batches and cross-contamination can readily result in false positive results when accurate quantification is required [15]. Recently, emerging technologies, including surface-enhanced Raman spectroscopy [16], electrochemical immunosensors [17], visualized microarrays [18], and transient isochronous electrophoresis [19], have been developed. However, the sample preparation process of these approaches can be cumbersome, and external environmental factors can easily influence the assay's results. Therefore, it is crucial to develop a rapid, accurate, and effective analytical approach for monitoring β-Lg levels in milk and dairy products to contribute to the standardization of food package labeling and protect public health.

Nucleic acid aptamers (Apt) are DNA or RNA oligonucleotides obtained via the systematic evolution of the exponentially enriched ligand approach [20]. They offer several advantages, such as low cost, good stability, specificity, and affinity compared with traditional antibodies. Furthermore, the primary advantage of using an aptamer is that there are no target limits. Although antibody-based assays are well established, they have limitations in recognizing a wide range of targets, including hazardous small compounds and nonimmunogenic targets. They are also slow in detecting minute variations in large molecular targets, such as proteins. Therefore, aptamers can be employed as outstanding biological recognition elements to develop various aptamer-based bio-sensors for detecting small molecules, proteins, and even cells [21,22,23]. Recently, several aptamer-based biosensors have been developed using different techniques such as fluorescence [24], chemiluminescence [25], electrochemistry [26], surface plasmon resonance [27], and colorimetry [28]. Among these techniques, fluorescent biosensors have gained significant attention due to their simplicity, rapidity, cost-effectiveness, and high sensitivity. Layered tungsten disulfide (WS_2_) nanosheets are transition metal dichalcogenides with an S-W-S intercalation structure. They have a high specific surface area, excellent photothermal conversion efficiency, good biocompatibility, and superior electrical properties. Furthermore, WS_2_ nanosheets have significantly higher fluorescence quenching efficiency compared with graphene oxide and can accurately identify single-stranded DNA (ssDNA) and double-stranded DNA (dsDNA), which is crucial for the sensitivity of quench- and recovery-based fluorescent aptamer biosensors [29]. Due to these advantages, WS_2_ has been successfully employed for detecting various substances. For instance, a new type of fluorescent aptamer biosensor based on up-conversion nanoparticles and WS_2_ nanosheets has been developed for detecting Escherichia coli [30]. Similarly, based on the high fluorescence quenching efficiency of WS_2_ nanosheets, effective fluorescent biosensors have been developed for detecting zearalenone [31] and kanamycin [32]. Therefore, WS_2_ nanosheets are an ideal material for constructing fluorescent aptamer biosensors to detect β-Lg.

**Figure 1:**
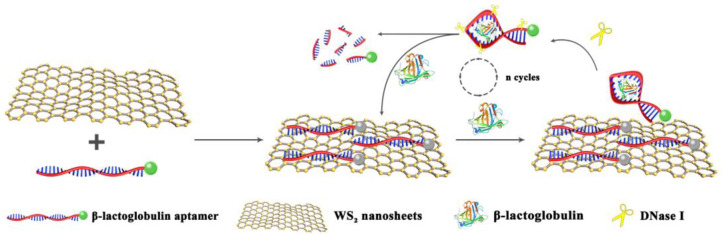
Working principle of the fluorescent aptamer biosensor for β-Lg detection.

In real food samples, the content of the target molecule is usually low, so a signal amplification strategy is necessary to enhance the sensitivity of the biosensor. Researchers have successfully applied various signal amplification methods in biosensors, including those employing catalytic and self-propagating cascade reactions. Rolling cycle amplification, catalytic hairpin assembly, strand displacement reaction, and hybridization chain reaction are also widely used by researchers [33,34,35]. Deoxyribonuclease I (DNase I) is frequently used as a nucleic acid endonuclease that can cleave ssDNA for aptamer biosensors [36]. DNase I can also cleave the aptamer/target complex in the detection system and release the target into the solution for recycling, thus providing a fluorescence amplification effect. For instance, a DNase I-assisted fluorescence signal amplification strategy was developed to reduce the quantitative limit of the reaction in Patulin [37] and prostate-specific antigen detection [38]. In this study, we developed a WS_2_ nanosheet-aptamer fluorescent biosensor based on fluorescein-based dye (FAM)-labeled β-lactoglobulin aptamer (β-Lg aptamer) by combining the enzymatic cycle amplification reaction of DNase I and the selective binding ability as well as the fluorescence quenching effect of WS_2_ nanosheets. In this sensing system, in the absence of the β-Lg, the β-Lg aptamer is adsorbed onto the WS_2_ nanosheet due to the van der Waals forces between the DNA nucleobase and the basal plane of the WS_2_ nanosheet [39,40], which results in a fluorescence resonance energy transfer phenomenon, leading to fluorescence quenching. However, in the presence of β-Lg, the β-Lg aptamer preferentially binds to β-Lg, resulting in a change in the aptamer’s conformation and weakening the interaction between the β-Lg aptamer and WS_2_ nanosheets. This releases the aptamer from the surface of WS_2_ nanosheets and restores fluorescence. At this time, DNase I present in the sensing system digests the fluorescent probe bound to the target, releasing β-Lg and generating a short oligonucleotide fragment. The cleaved FAM-labeled oligonucleotide fragment does not adsorb into the WS_2_ nanosheet due to its weak affinity with the WS_2_ nanosheet and thus retains a strong fluorescent signal. Meanwhile, β-Lg continued to bind to another fluorescent probe adsorbed on WS_2_ nanosheets and initiates the next round of cleavage after releasing the target β-Lg, resulting in significant amplification in the fluorescence signal. The change in fluorescence signal intensity detects the β-Lg content (Figure 1). This approach is highly specific and sensitive and has been demonstrated to be useful for practical sample detection.

## 2. Results

### 2.1. Feasibility Study of the Fluorescent Biosensor

**Figure 2:**
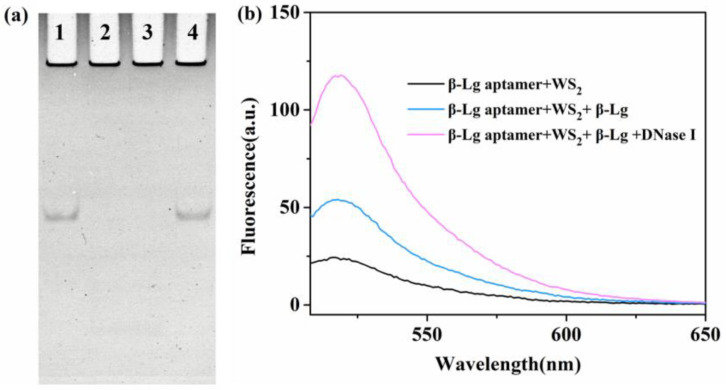
(**a**) Gel electrophoresis of the β-Lg aptamer. Lane 1: β-Lg aptamer; lane 2: β-Lg aptamer + DNase I; lane 3: β-Lg aptamer + β-Lg + DNase I; lane 4: β-Lg aptamer + WS_2_ + DNase I. (**b**) Fluorescence spectra of β-Lg aptamer in different cases: (black line) β-Lg aptamer + WS_2_; (blue line) β-Lg aptamer + WS_2_ + β-Lg; (pink line) β-Lg aptamer + WS_2_ + β-Lg + DNase I.

To verify the feasibility of the enzymatic cycle amplification strategy, we conducted gel electrophoresis analyses using β-Lg and a β-Lg aptamer as models. After incubating the β-Lg aptamer with DNase I, it was completely cleaved (Figure 2a, lane 2). DNase I could also cleave the β-Lg aptamer in the presence of β-Lg (Figure 2a, lane 3). However, the β-Lg aptamer adsorbed on WS_2_ nanosheets showed no evident enzymatic hydrolysis, indicating its resistance to enzymatic digestion (Figure 2a, lane 4). To further determine the feasibility of the fluorescent biosensor for β-Lg detection, Figure 2b illustrates the fluorescence emission spectra of the β-Lg aptamer under different conditions. When WS_2_ nanosheets were introduced into the system, the aptamer was easily adsorbed on the WS_2_ surface due to the van der Waals force between the strong β-Lg aptamer base and WS_2_ nanosheets. Furthermore, WS_2_ nanosheets have broad absorption ranging from 200 to 800 nm (Figure S1 in Supplementary Materials), and the β-Lg aptamer’s fluorescence emission spectra overlapped well, resulting in FRET and a substantial decrease in fluorescence intensity. The high specificity between the β-Lg and β-Lg aptamer in the presence of β-Lg caused the aptamer to be released from the WS_2_'s surface and formed a β-Lg-aptamer complex, resulting in fluorescence recovery (blue line). To further enhance sensitivity, we employed an enzymatic cycle amplification approach. The β-Lg-aptamer complex was formed when the system contained the targeted β-Lg. Meanwhile, DNase I could cleave the β-Lg aptamer, releasing β-Lg and liberating the FAM fluorophore. Then, the released β-Lg could bind to another β-Lg aptamer, initiating the next round of cleavage and leading to significant fluorescence recovery compared to the system without DNase I. Due to this advantage, upon adding DNase I, fluorescence increased by approximately 3-fold, serving the purpose of signal amplification (pink line). These results confirm the feasibility of the proposed fluorescence amplification method.

### 2.2. Optimization of the Testing Conditions

**Figure 3:**
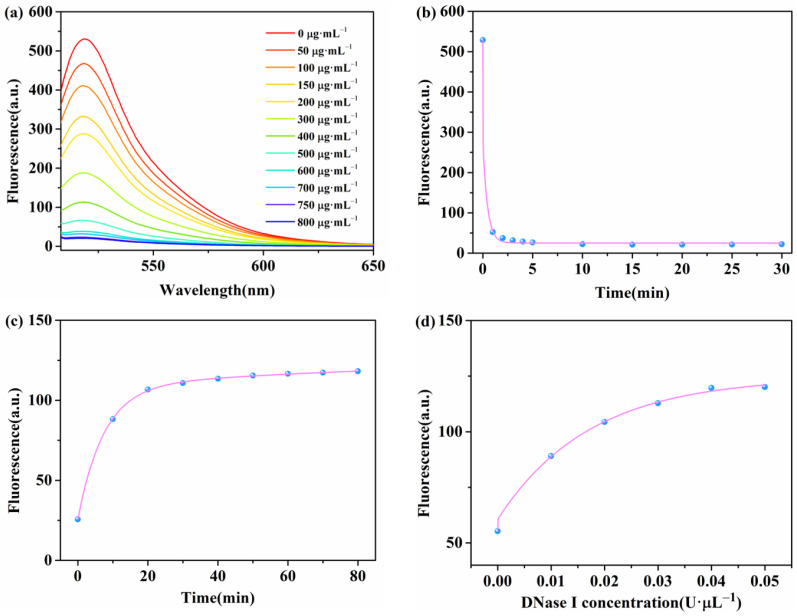
(**a**) Fluorescence spectra of β-Lg aptamer after adding different concentrations (from a to l: 0, 50, 100, 150, 200, 300, 400, 500, 600, 700, 750, and 800 μg·mL^−1^) of WS_2_ nanosheets. (**b**) Fluorescence intensity of β-Lg aptamer in response to various times. (**c**) Effect of recovery time on the fluorescence response of the β-Lg (10 μg·mL^−1^) detection system. (**d**) Effect of enzyme concentration on the fluorescence response of the β-Lg (10 μg·mL^−1^) detection system.

Several important parameters were optimized to obtain the best fluorescence signal. These included the concentration of WS_2_ nanosheets, the quenching time of the method, the fluorescence recovery time of the system, and the concentration of DNase I. First, the concentration of WS_2_ nanosheets was optimized to enhance the sensing performance. The fluorescence intensity of the β-Lg aptamer decreased substantially with the increasing concentration of WS_2_ nanosheets, while the concentration of β-Lg aptamer remained constant (Figure 3a). The fluorescence quenching efficiency reached 96% when the concentration of WS_2_ nanosheets increased to 750 μg·mL^−1^, demonstrating that WS_2_ nanosheets can be an effective quencher. A further increase in the concentration of WS_2_ nanosheets did not result in any significant changes in fluorescence intensity. Therefore, WS_2_ nanosheets at a concentration of 750 μg·mL^−1^ were chosen for subsequent experiments to achieve the required sensitivity and selectivity of the assay. Subsequently, we analyzed the quenching time of the method as well as the fluorescence recovery time of the reaction system. Figure 3b illustrates the experimental results. The fluorescence intensity decreased rapidly and reached equilibrium in about 10 min when conducting WS_2_ with the β-Lg aptamer (Figure 3c). Therefore, we considered 10 min as the optimal quenching time. With the addition of β-Lg and DNase I, the fluorescence intensity gradually increased over time, rapidly increasing within 40 min and then slowly increasing from 40 to 80 min. Accordingly, we selected 40 min as the optimal incubation time for β-Lg and DNase I. Finally, we optimized the concentration of DNase I by adding varying concentrations of DNase I (0, 0.01, 0.02, 0.03, 0.04, and 0.05 U·μL^−1^) to the reaction system while keeping the other conditions consistent. The fluorescence intensity increased with increasing enzyme concentration and reached saturation at 0.04 U·μL^−1^ (Figure 3d). Therefore, we used this concentration of DNase I in subsequent assays.

### 2.3. Sensitivity of the Aptamer Biosensor

**Figure 4:**
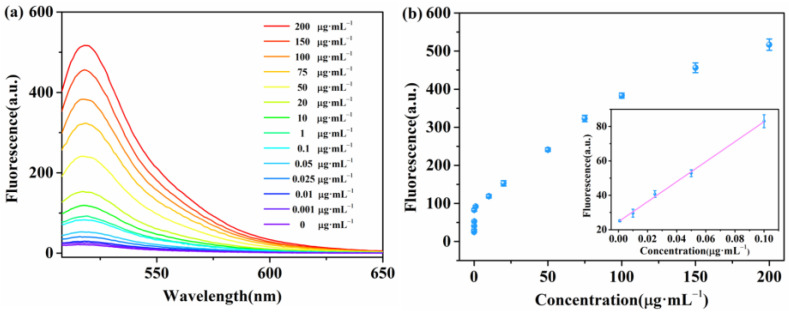
(**a**) Fluorescence emission spectra of β-Lg aptamer at different concentrations of β-Lg (from a to n: 0, 0.001, 0.01, 0.025, 0.05, 0.1, 1, 10, 20, 50, 75, 100, 150, and 200 μg·mL^−1^). (**b**) The standard curve of β-Lg. Insert: linear fitting of fluorescence intensity versus target β-Lg concentration at 518 nm.

**Figure 5:**
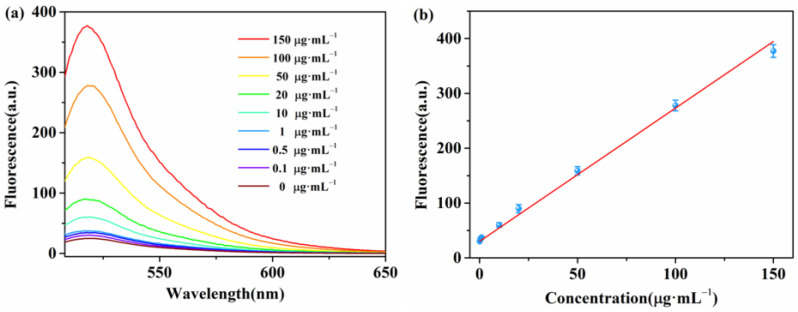
(**a**) Fluorescence emission spectra of β-Lg aptamer at various concentrations of β-Lg (from a to i: 0, 0.1, 0.5, 1, 10, 20, 50, 100, and 150 μg·mL^−1^). (**b**) The standard curve of β-Lg. Insert: linear fitting of fluorescence intensity versus target β-Lg concentration at 518 nm.

We measured different concentrations of β-Lg under the optimal experimental conditions described above to analyze the sensitivity of the proposed fluorescent biosensor. We added a series of concentrations (0, 0.001, 0.01, 0.025, 0.05, 0.1, 1, 10, 20, 50, 75, 100, 150, and 200 μg·mL^−1^) of β-Lg to the proposed detection system. The emission peaks were recorded at 518 nm. As shown in Figure 4a, the fluorescence intensity at 518 nm increased with an increase in β-Lg concentration from 0 to 200 μg·mL^−1^, indicating the high dependence of the biosensor on the target concentration in detecting β-Lg. A good linear relationship was observed between the fluorescence intensity and the concentration of β-Lg within the range of 1–100 ng mL^−1^ (Figure 4b). The calibration function is *F* = 581.5*C* + 24.6 (*R*^2^ = 0.9969), where *C* represents the concentration of β-Lg, and *F* represents the fluorescence intensity. Furthermore, we calculated the limit of detection of the method as 0.344 ng mL^−1^ using equation 3*S*_0_/*K*, where *S*_0_ is the standard deviation of the blank test (n = 10), and *K* is the slope of the calibration curve. We also established the analytical performance for detecting β-Lg without DNase I (Figure 5). The fluorescence intensity at 518 nm increased with an increase in β-Lg concentrations from 0.1 to 150 μg·mL^−1^. The detection limit was determined to be 35 ng mL^−1^. The sensitivity of the proposed fluorescent biosensor was more than two orders of magnitude higher than that of unamplified fluorescent biosensor assays. A series of five repetitive measurements with the same concentrations were used to investigate the precision of the proposed method. The relative standard deviations for 1 ng mL^−1^ and 10 ng mL^−1^ of β-Lg were 2.44% and 4.43%, respectively, indicating good reproducibility of the assay. Currently, there are few studies detailing the use of DNA aptamers for β-Lg-detection-exploiting enzymes, and a comparison with other β-Lg assays is provided in Table 1. The approach demonstrated similar or better analytical performance compared with other β-Lg assays. The high sensitivity of the aptamer biosensor based on WS_2_ nanosheets and DNase I indicated its potential for detecting β-Lg in food samples.

### 2.4. Specificity and Stability of the Aptamer Biosensor

**Figure 6:**
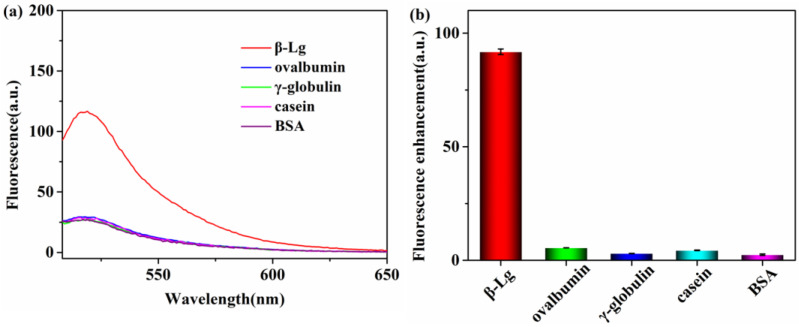
Selectivity of the method. (**a**) Fluorescence emission spectra of the system in the presence of different proteins. (**b**) Fluorescence changes of the system toward β-Lg and other interfering proteins.

Another important factor that affects the performance of an analytical assay is its specificity toward the targeted analyte. We evaluated the specificity of the method using two different approaches. First, we selected some other proteins, namely ovalbumin, γ-globulin, casein, and BSA (10 μg·mL^−1^), as interfering proteins. The presence of these proteins did not significantly restore the fluorescence intensity of the β-Lg aptamer compared with β-Lg, as demonstrated in Figure 6. For the second approach, we used a negative control DNA sequence to adsorb onto the WS_2_ nanosheet and evaluated the fluorescence change in the presence of different β-Lg concentrations (0.1, 1, 10, and 100 μg·mL^−1^). Little fluorescence changes were observed in the presence of the negative control DNA sequence, independent of the concentration of β-Lg (Figure S2). These findings demonstrate that the designed fluorescent biosensor has high selectivity for β-Lg. To verify the stability of the proposed aptamer biosensor, we stored the β-Lg aptamer/WS_2_ complex at 4 °C and measured its response to 10 μg·mL^−1^ of β-Lg every two days. The β-Lg aptamer could still recover its fluorescence signal in the β-Lg aptamer/WS_2_ complex and retained 90.8% of its initial fluorescence signal after two weeks (Figure S3). This result demonstrates that the quenching efficiency of WS_2_ nanosheets for the β-Lg aptamer was stable.

### 2.5. Actual Sample Testing

Finally, to further examine the potential application of fluorescent biosensors in food samples, we used milk and infant formula samples for the assay. As indicated in Table 2, the testing results of β-Lg in milk samples obtained using the proposed fluorescent biosensors and HPLC were similar. The t-test values for the correlation were less than 2.78 (the t-test value was 2.78 at a 95% confidence level). Since no β-Lg was detected in the infant formula samples, the recovery rates of β-Lg were determined using a standard addition approach. We added known concentrations (1, 10, and 100 ng·mL^−1^) of the β-Lg standard solution to the detection system. The recovery rates of β-Lg ranged from 94.3% to 99.5% for the infant formula samples, as shown in Table 3. These application's findings demonstrate that the established fluorescent biosensor has excellent accuracy and a high potential for quantifying β-Lg in real complex matrices.

## 3. Materials and Methods

### 3.1. Materials and Reagents

DNase I was purchased from New England Biolabs (Beijing, China), Ltd., and WS_2_ nanosheets were obtained from Nanjing XFNANO Materials Tech Co., Ltd. (Nanjing, China). The β-Lg aptamer (5′-FAM-CGACGATCGGACCGCAGTACCCACAGCCCCAACATCATGCCCATCCGTGTGTG-3′) [43] and the negative control DNA sequence (5′-FAM-CTAAGTCTGAAACATTACAGCTTGCTACACGAGAAGAGCCGCACGAGAACCCT) were synthesized by Shanghai Sangon Biotechnology Co., Ltd. (Shanghai, China). β-Lg, ovalbumin, and casein were purchased from Sigma Aldrich Chemical Co., Ltd. (St. Louis, MO, USA). The milk samples and infant formula samples were purchased from supermarkets in Xiamen, China. Other reagents were of analytical grade and were used without further purification. All solutions were prepared and diluted using ultrapure water (18.2 MΩ cm) produced by the Millipore Milli-Q system.

### 3.2. Apparatus

The fluorescence measurements were recorded using an LS 55 fluorescence spectrometer (PerkinElmer Ltd., Waltham, MA, USA). Ultraviolet–visible–near-infrared light (UV-Vis-NIR) absorption spectrums were recorded using a Lambda 265 UV-vis spectrophotometer (PerkinElmer Ltd., USA). The pH measurements were conducted using a digital PE 28 pH meter (Mettler Toledo, Greifensee, Switzerland).

### 3.3. Optimization of the Experimental Conditions

First, we optimized the concentration of WS_2_ nanosheets. We added various concentrations of WS_2_ nanosheets (0–800 μg·mL^−1^) to a fixed concentration of β-Lg aptamer (200 nM) and incubated it for 10 min at room temperature to determine the optimal quenching concentration. Next, we optimized the fluorescence quenching time (0, 1, 2, 3, 4, 5, 10, 15, 20, 25, and 30 min) and recovery time (0, 10, 20, 30, 40, 50, 60, 70, and 80 min) under the above optimal conditions based on the fluorescence emission intensity. Furthermore, to determine the optimized concentration of DNase I, we added different concentrations of DNase I (0, 0.01, 0.02, 0.03, 0.04, and 0.05 U·μL^−1^) to the β-Lg aptamer/WS_2_ detection system and incubated it for 40 min at 37 °C. The excitation wavelength was 490 nm, and the fluorescent spectra were measured between 500 and 650 nm. The fluorescence emission intensity was recorded at 518 nm. The fluorescence quenching efficiency (*Q_E_*) was computed using Equation (1):

(1) $$Q_{E}=\frac{F_{0}-F}{F_{0}}$$

where *F*_0_ represents the fluorescence intensity at 518 nm in the absence of WS_2_ nanosheets, and *F*_q_ represents the fluorescence intensity at 518 nm in the presence of WS_2_ nanosheets.

### 3.4. Detection of β-Lg

In a typical fluorescence experiment, a mixture of 150 μL of the β-Lg aptamer (200 nM) and 150 μL of WS_2_ nanosheets (750 μg·mL^−1^) was gently shaken for 10 min at room temperature. Then, β-Lg at various concentrations (0, 0.001, 0.01, 0.025, 0.05, 0.1, 1, 10, 20, 50, 75, 100, 150, and 200 μg·mL^−1^) in 141 µL of reaction buffer (50 mM Tris-HCl, 150 mM NaCl, 2 mM MgCl_2_, and pH 7.5) and DNase I (2000 U·mL^−1^) in 9 μL were added to the above solution at 37 °C for 40 min to initiate signal amplification. The fluorescent spectra were measured using the above procedure.

### 3.5. Selectivity Experiment

For the assay of interfering proteins, the β-Lg aptamer/WS_2_ detection system was mixed with 10 μg·mL^−1^ of β-Lg, ovalbumin, γ-globulin, casein, and BSA. The fluorescent spectra were measured according to the above procedure.

### 3.6. Stability and Reproducibility Experiment

To assess the stability of the biosensor, the β-Lg aptamer/WS_2_ complex was stored at 4 °C for two weeks. Every two days, 300 µL of the complex solution was added to 141 µL of β-Lg solution (10 μg·mL^−1^) and 9 µL of DNase I (2000 U·mL^−1^). The resulting mixture solution was incubated at 37 °C for 40 min, and then the fluorescence spectra were measured. To demonstrate the reproducibility of the method, the fluorescence intensity of the biosensor was measured for the same concentration of β-Lg (10 μg·mL^−1^) under optimal conditions on five consecutive occasions.

### 3.7. Detection of β-Lg in Actual Samples

The actual samples included milk and infant formula samples. These samples were pretreated according to previous reports [44]. For milk samples, the following steps were followed: The samples were heated at 40 °C for 30 min and then centrifuged at 8000 rpm for 20 min. After that, the samples were cooled for 15 min. The supernatant was collected, and the pH was adjusted to 4.6. The supernatant was further centrifuged at 8000 rpm for 20 min to precipitate casein and filtered through a 0.2 μm polycarbonate membrane. Then, the filtered solution was adjusted to pH 7.5 with 1 M NaOH. Finally, the pretreated milk samples were diluted to produce concentrations within the linear detection range. For infant formula samples, milk powder (8 g) was dissolved in 40 mL ultrapure water, and the remaining steps were the same as for the milk samples. β-Lg solutions with different concentrations (1, 10, and 100 ng·mL^−1^) were diluted with the above solution, and detecting β-Lg in the actual samples was conducted according to the steps of the β-Lg assay.

## 4. Conclusions

In this study, we presented a highly sensitive β-Lg fluorescent aptamer biosensor based on WS_2_ nanosheets and DNase I-assisted cyclic signal amplification approach. The sensitive detection performance of this biosensor can be easily achieved by combining the β-Lg aptamer/WS_2_ complex, DNase I, and target β-Lg. This approach takes advantage of the strong quenching ability of WS_2_ and the unique function of DNase I to release FAM-labeled short oligonucleotide fragments via a cyclic cleavage process, resulting in a significant amplification of the fluorescence signal. The sensing system demonstrates excellent selectivity for β-Lg, with a limit of detection of 0.344 ng·mL^−1^, which is around two orders of magnitude higher than that of unamplified fluorescent biosensor assays. In summary, the proposed biosensor offers a simple and convenient alternative to standard approaches for food monitoring and demonstrates an ultrasensitive detection of β-Lg.

## Figures and Tables

**Table 1:** Comparison of different β-Lg assays.

Methods	Linear Range (μg·mL^−1^)	LOD (μg·mL^−1^)	References
QCM immunochip	0.5–1000	0.04	[8]
LC-MS/MS	0.48–31.25	0.2	[11]
UPLC	20–560	7	[12]
Sandwich ELISA	0.03125–8	1.96 × 10^−3^	[14]
Fluorescence sensor	100–800	43	[15]
Surface Plasmon Resonance	0.49–1000	0.164	[41]
Electrochemical aptasensor	1 × 10^−4^–0.01	9 × 10^−5^	[42]
Fluorescence aptamer biosensor	1 × 10^−3^–0.1	3.44 × 10^−4^	This work

**Table 2:** Determination of β-Lg in milk samples (n = 3) using different methods.

Milk Samples	Detected Concentration (mg·mL^−1^)		*t*-Test
	This Work	HPLC	
1	3.48 ± 0.16	3.39 ± 0.04	0.92
2	3.53 ± 0.17	3.62 ± 0.08	0.82
3	3.90 ± 0.09	4.07 ± 0.07	2.60

*t*_table_ = 2.78, *p* = 0.95, and *v* = 4.

**Table 3:** Determination of β-Lg in infant formula.

Samples	β-Lg Spiked (ng·mL^−1^)	β-Lg Found (ng·mL^−1^)	Recovery (%)	RSD (%)
Infant formula	-	Not detected	-	-
	1	0.978	97.8	3.8
	10	9.95	99.5	3.3
	100	94.31	94.3	5.4

## Data Availability

Not applicable.

## Supplementary Materials

The following supporting information can be downloaded at https://www.mdpi.com/article/10.3390/molecules28083502/s1. Figure S1. UV–Vis absorption spectra and TEM image of WS_2_ nanosheets; Figure S2. The selectivity of the method using negative control DNA sequence; Figure S3. The stability of β-Lg aptamer/WS_2_ complex for β-Lg detection.

## Sample Availability

Samples of the compounds are available from the companies listed.
