# Supplementary material for: Highly Sensitive β-Lactoglobulin Fluorescent Aptamer Biosensors Based on Tungsten Disulfide Nanosheets and DNase I-Assisted Signal Amplification
Source: Molecules. 2023 Apr 16;28(8):3502. doi: 10.3390/molecules28083502 (PMC10146092; doi:10.3390/molecules28083502)
Supplement: Supplementary file 1 [file molecules-28-03502-s001.zip › molecules-2271979-supplementary.pdf]

Supporting Information

Highly Sensitive β-Lactoglobulin Fluorescent Aptamer Biosensors Based on Tungsten Disulfide Nanosheets and DNase I-Assisted Signal Amplification

Yuying Wang [1], Sisi Chen [1], Wanmei Chen [1], Jingjing Wang [1], Kun Li [2], Chengyi Hong [1,3*], Kailong Zhang [2,4,5*], and Quansheng Chen [1]

1 College of Ocean Food and Biological Engineering, Jimei University, Xiamen 361021, China;

2 School of Life Sciences, Longyan University, Longyan 364012, China;

3 Fujian Provincial Key Laboratory of Food Microbiology and Enzyme Engineering, Xiamen 361021, China;

4 Fujian Provincial Key Laboratory for the Prevention and Control of Animal Infectious Diseases and Bio-technology, Longyan 364012, China;

5 Fujian Province Universities Key Laboratory of Preventive Veterinary Medicine and Biotechnology (Longyan University), Longyan 364012, China;

* Corresponding author.

Email: cyhong@jmu.edu.cn; klzhang@lyun.edu.cn.

Supporting Figure

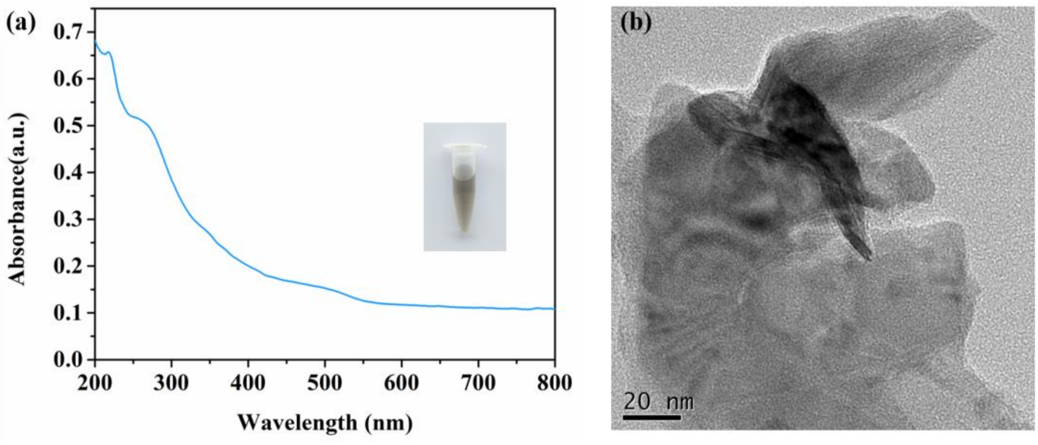

Figure S1. (a) UV–Vis absorption spectra and corresponding photographs of WS_2 nanosheets. (b) TEM image of the WSe_2 nanosheets.

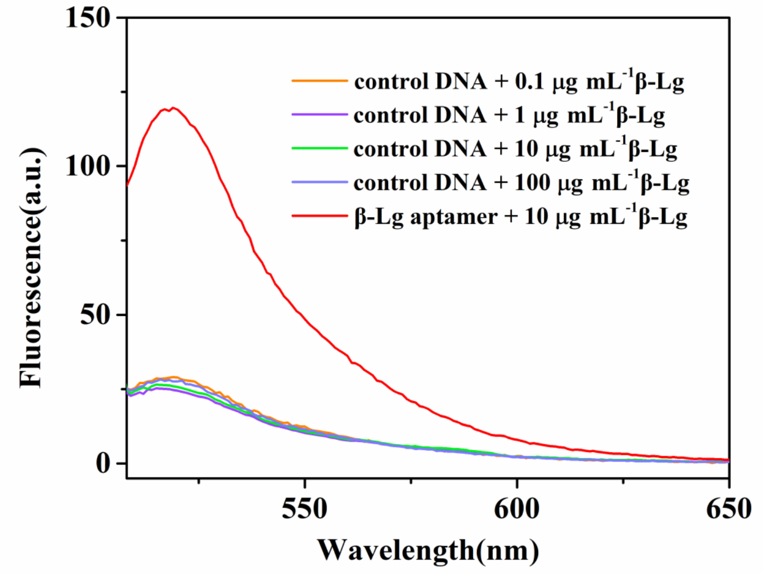

Figure S2. Fluorescence spectra of β-Lg aptamer in different cases: β-Lg aptamer+WS_2+β-Lg+ DNase I and control DNA sequence+WS_2+β-Lg+ DNase I.

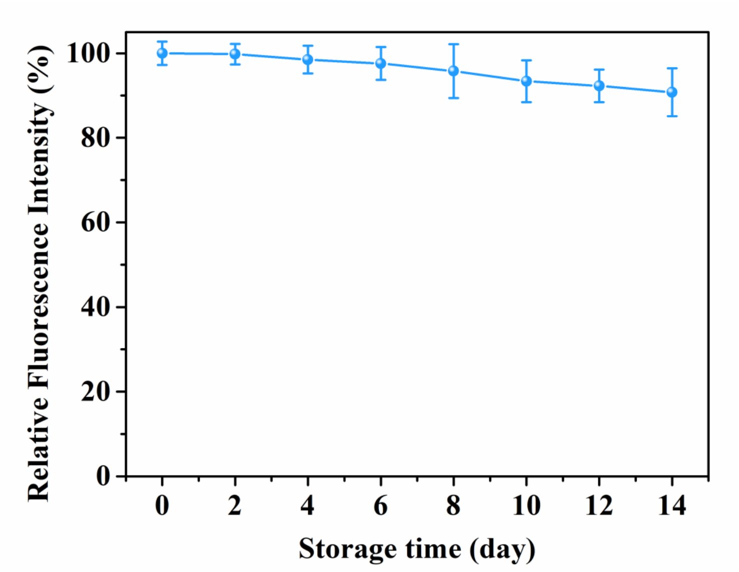

Figure S3. The stability of β-Lg aptamer/WS_2 complex for β-Lg detection.
